# DNA binding and transposition activity of the Sleeping Beauty transposase: role of structural stability of the primary DNA-binding domain

**DOI:** 10.1093/nar/gkae1188

**Published:** 2024-12-09

**Authors:** Venkatesh V Ranjan, Gage O Leighton, Chenbo Yan, Maria Arango, Janna Lustig, Rosario I Corona, Jun-Tao Guo, Yuri E Nesmelov, Zoltán Ivics, Irina V Nesmelova

**Affiliations:** Department of Chemistry, University of North Carolina at Charlotte, 9201 University City Blvd., Charlotte, NC 28223, USA; Department of Physics and Optical Science, University of North Carolina at Charlotte, 9201 University City Blvd., Charlotte, NC 28223, USA; Department of Physics and Optical Science, University of North Carolina at Charlotte, 9201 University City Blvd., Charlotte, NC 28223, USA; Department of Biochemistry and Biophysics, University of North Carolina at Chapel Hill, 120 Mason Farm Rd., Chapel Hill, NC 27599, USA; Department of Physics and Optical Science, University of North Carolina at Charlotte, 9201 University City Blvd., Charlotte, NC 28223, USA; Department of Physics and Optical Science, University of North Carolina at Charlotte, 9201 University City Blvd., Charlotte, NC 28223, USA; Division of Hematology, Gene and Cell Therapy, Paul Ehrlich Institute, Paul Ehrlich Strasse 51-59, 63225 Langen, Germany; Department of Bioinformatics and Genomics, University of North Carolina at Charlotte, 9201 University City Blvd., Charlotte, NC 28223, USA; Department of Bioinformatics and Genomics, University of North Carolina at Charlotte, 9201 University City Blvd., Charlotte, NC 28223, USA; Department of Physics and Optical Science, University of North Carolina at Charlotte, 9201 University City Blvd., Charlotte, NC 28223, USA; Division of Hematology, Gene and Cell Therapy, Paul Ehrlich Institute, Paul Ehrlich Strasse 51-59, 63225 Langen, Germany; Department of Physics and Optical Science, University of North Carolina at Charlotte, 9201 University City Blvd., Charlotte, NC 28223, USA; School of Data Science, University of North Carolina at Charlotte, Charlotte, 9201 University City Blvd., NC 28223, USA

## Abstract

DNA transposons have emerged as promising tools in both gene therapy and functional genomics. In particular, the Sleeping Beauty (SB) DNA transposon has advanced into clinical trials due to its ability to stably integrate DNA sequences of choice into eukaryotic genomes. The efficiency of the DNA transposon system depends on the interaction between the transposon DNA and the transposase enzyme that facilitates gene transfer. In this study, we assess the DNA-binding capabilities of variants of the SB transposase and demonstrate that the structural stability of the primary DNA-recognition subdomain, PAI, affects SB DNA-binding affinity and transposition activity. This fundamental understanding of the structure–function relationship of the SB transposase will assist the design of improved transposases for gene therapy applications.

## Graphical Abstract

**Graphical Abstract:**
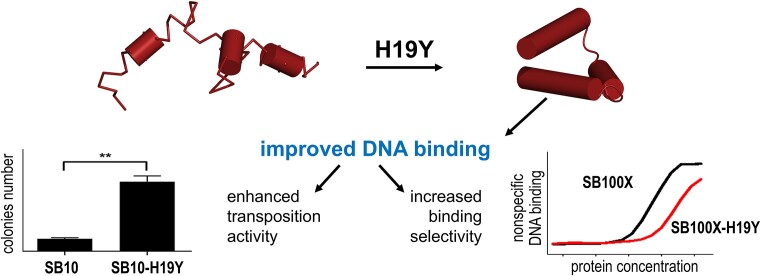

## Introduction

The use of DNA transposons as a means of delivering new genetic information into vertebrate organisms and cells (1,2) relies on their ability to relocate from one DNA site to another (3,4). Specifically, when a transposition reaction is exploited to insert foreign genes into cells, relocation of a gene of interest typically occurs between a plasmid, which carries the transposon, and a genome. DNA transposon-based technologies have become powerful tools for functional genomics, transgenesis and insertional mutagenesis (5–7). Moreover, the ‘Sleeping Beauty’ (SB) and ‘piggyBac’ transposon systems are in clinical trials to test their potential to correct genetic disease (8–11). Recent advances in understanding SB’s mechanism of action have enabled the improvement of its safety for gene therapy by directing the transposon away from transcriptional regulatory regions and exons (12).

**Figure 1.**
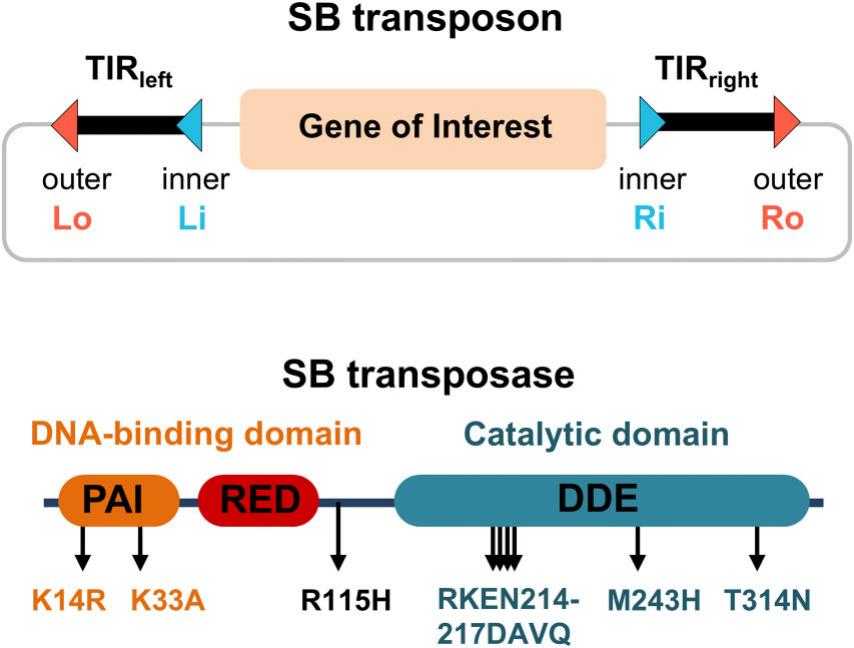
The schematic presentation of SB transposon (top panel) and SB transposase (bottom panel) structures. SB transposon consists of the gene of interest to be delivered, flanked by TIRs (TIR_left_ and TIR_right_), each containing two (inner and outer) transposase-binding sites. SB transposase consists of the catalytic domain and the DNA-binding domain containing two subdomains, PAI and RED. The locations of hyperactive mutations are indicated by arrows.

A DNA transposon gene delivery system typically consists of two essential components: a transposon DNA containing the gene of interest flanked by terminal inverted repeats (TIRs) and a transposase enzyme that facilitates the transfer of the gene (13–19) (Figure 1). In the case of SB, the TIRs contain four imperfect 32-bp direct repeats (DRs). The outer DRs are situated at the ends of the transposon, while the inner DRs are located inside the transposon 165- to 166-bp away from the outer DRs (14,20). SB transposition begins with site-specific binding of the SB transposase to the DRs (Figure 1). These DRs contain a shared 18-bp DR-core sequence, while the surrounding adjacent sequences vary among the four transposase-binding sites, with this variation being significant for efficient transposition (21). Each DR serves as a binding site for the transposase (21). The efficiency of a DNA transposon system depends in part on how well the transposon and the transposase enzyme work together, which has prompted modifications to both the transposon and the transposase to achieve higher transposition rates (12,15,22). Using molecular evolution approaches, specific mutations in both the piggyBac and SB transposases have been shown to support ∼17- and ∼100-fold increases of transposition rates, respectively (15,19).

While molecular evolution is a powerful tool for improving transposase activity, it does not provide insights into the underlying molecular mechanisms because mutations leading to hyperactivity are distributed throughout the entire protein. The SB transposase is a multidomain protein that consists of an N-terminal DNA-binding domain and a C-terminal catalytic domain (Figure 1). The DNA-binding domain is further divided into the PAI and RED subdomains (collectively called the paired domain, PAI + RED = paired), which are connected by a flexible linker (14,23). In the most active variant of the SB transposase, SB100X, the hyperactive mutations include K14R and K33A mutations in PAI, RKEN214-217DAVQ, M243H and T314N in the catalytic domain, and R115H mutation in the linker between the catalytic and DNA-binding domains (15). K33A increases DNA-binding activity (24), and K14R results in higher transposition rates without changing DNA-binding affinity (15). Crystallographic structural analysis has revealed the basis of several mutations that increase overall catalytic activity of SB100X transposase (PDB code 5cr4). The data suggest that the substitution T314N located on the protein surface increases its solubility (25) and C176S and I212S substitutions are more able to penetrate cellular membranes, thereby enabling direct protein administration (26). The SB100X crystallographic structure further suggests that the M243H substitution likely assists in positioning the catalytic residue D244 in the active site, while the RKEN214-217DAVQ mutations, which immediately follow the loop that participates in DNA-binding, likely aid in shaping and optimally positioning this loop for DNA interactions (25). Furthermore, RKEN214-217DAVQ mutations favorably alter mechanical couplings within the SB transposase (27).

The above studies show the importance of understanding transposase–transposon-binding structure to enable better use of the SB system for its many roles in research and gene therapy. Previously, we have solved the structure of PAI (28) and demonstrated that it selectively binds to the transposon DNA through a conformational selection mechanism (29). This means that PAI’s interaction with DNA is contingent upon its pre-existing conformation and only those molecules that exist in a DNA-binding-competent state can initiate binding. To test this hypothesis, we engineered a structurally stable variant of PAI with enhanced DNA-binding capabilities by introducing the H19Y mutation to better understand the structure of the PAI–transposon DNA complex and gain mechanistic insights into the PAI–DNA-binding process. We show that increased structural stability of PAI associated with the H19Y mutation correlated with enhanced transposition activity of the SB10 transposase and with increased DNA-binding selectivity of SB100X transposase.

## Materials and methods

### Protein expression, purification and sample preparation

The nomenclature of proteins used in this study is as follows: PAI (PAI from SB10 transposase without modifications), PAI-K14RK33A (PAI with mutations K14R and K33A), H19Y (PAI with three mutations K14R, H19Y and K33A), SB10 [the first-generation full-length SB10 transposase as reported in (14)], SB10-H19Y (SB10 with H19Y mutation), SB100X transposase [full-length 100-times more active SB transposase as reported in (15)] and SB100X-H19Y (SB100X transposase with H19Y mutation). For reference, amino acid sequences of all proteins are provided in Supplementary Table S1. All proteins were expressed and purified following a previously reported protocol (28). DNA plasmids encoding proteins were ordered from GenScript USA, Inc. The proteins were expressed in BL21‐AI *Escherichia coli* cells. For ^15^N and ^13^C isotopic labeling, bacterial cells were grown in M9 medium using ^15^NH_4_Cl and ^13^C‐glycerol (Cambridge Isotope Laboratories) as the sole nitrogen and carbon sources, respectively. The proteins were purified by metal chelating chromatography using a Ni‐NTA Agarose (Thermo Fisher Scientific). Samples with PAI subdomain or its variants were prepared in an aqueous 25 mM sodium phosphate buffer at pH 5.2 or pH 7.4. For nuclear magnetic resonance (NMR) experiments, the buffer contained 5% D_2_O. To assess the effect of pH on protein structure, the solution pH was increased from 4.5 to 8.5, in 0.5 increments, by adding NaOH. Purified SB10 and SB100X full-length transposases were prepared in an aqueous 50 mM Tris buffer at pH 7.5, containing 5 mM MgCl_2_, 300 mM NaCl, 2% glycerol, 50 mM Arg-Glu mixture and 1 mM TCEP (tris(2-carboxyethyl)phosphine). For DNA-binding experiments, unlabeled or Cy5-labeled DR-core, Li and Lo sequences were synthesized by IDT (Integrated DNA Technologies, Inc.). The Cy5 label was attached to the 5′ end of the forward DNA.

### Circular dichroism spectroscopy

Circular dichroism (CD) measurements were performed on a Jasco-715 spectropolarimeter, equipped with a Peltier temperature control system, using quartz glass cell with a path length, *l*, of 1 mm. Far-ultraviolet CD spectra were recorded in the range of 190–250 nm at room temperature. The corresponding buffer (25 mM sodium phosphate buffer) baseline was subtracted from each spectrum. Spectra were recorded using a 50 nm/min scan rate with a 4-s response and a 1-nm bandwidth. Reported spectra are averages of 2–5 scans and are expressed as mean residue ellipticity (MRE) calculated by using the following relation:


(1) \begin{equation*}MRE = \frac{{{M_0}{\theta _\lambda }}}{{100 \cdot C \cdot \lambda }},\end{equation*}

where *M_0_* is the mean residue molar mass, θ*_λ_* is the measured ellipticity in degrees and *C* is the total concentration of protein. The value of *M*_0_ was obtained by dividing the molecular weight of the protein with the number of amino acid residues in it. To follow the folding of PAI subdomain and its mutants, MRE at 222 nm, [θ]_222_, was used to assess protein structural changes.

CD spectra were analyzed on the DichroWeb server (30,31) to estimate the fraction of secondary structure elements. The dependence of the fraction of alpha-helical conformation, *f*_h_, on pH was fit to a modified Henderson–Hasselbalch equation:


(2) \begin{equation*}{f_{\rm h}} = \frac{{{f_{\rm a}} + {f_{\rm b}}\left( {{{10}^{n\left( {{\rm pKa} - {\rm pH}} \right)}}} \right)}}{{1 + {{10}^{n\left( {{\rm pKa} - {\rm pH}} \right)}}}}.\end{equation*}

In this equation, *f*_a_ is the fraction of alpha-helical conformation at acidic pH prior to transition, *f*_b_ is the fraction of alpha-helical conformation at basic pH after transition, pKa is the pH value corresponding to an inflection point of the dependence, and the *n* value (Hill coefficient) is the slope at the inflection point, which determines the number of protons involved in the transition. The Hill coefficient was set to be a free parameter during fitting.

NMR spectroscopy

#### NMR signal assignments and restraints acquisition

NMR experiments were performed on a Bruker Avance‐III 950- and 700-MHz spectrometers equipped with helium-cooled cryoprobes at David H. Murdock Research Institute and in the Molecular Education, Technology, and Research Innovation Center at North Carolina State University, respectively. NMR experiments for structure determination were carried out at 5°C, whereas DNA-binding experiments were carried out at 35°C. Proton chemical shifts were calibrated with respect to water signal relative to DSS [(CH_3_)_3_Si(CH_2_)_3_SO_3_Na] and ^15^N and ^13^C chemical shifts were indirectly referenced to DSS (32). Sequence‐specific resonance assignments have been performed using 3D HNCACB, CBCA(CO)NH, HCCH‐TOCSY, ^15^N‐separated TOCSY‐HSQC and HSQC‐NOESY, and ^13^C‐separated NOESY‐HSQC experiments as described in original references (33). Interproton distance restraints were derived from Nuclear Overhauser Enhancement (NOE) signals in ^15^N‐NOESY‐HSQC and ^13^C‐NOESY‐HSQC experiments, collected at 120 ms mixing time. Hydrogen bond restraints were identified from the pattern of sequential and inter‐helical NOEs involving NH and CαH protons and with evidence of slow amide proton‐solvent exchange, monitored with a series of 2D [^1^H,^15^N]‐HSQC spectra recorded in 100% D_2_O. All NMR spectra were processed using the NMRPipe software (34). Linear prediction was applied for both ^15^N and ^13^C dimensions to double the data size and improve the digital resolution, and cosine square window function and automatic zero filling were used. NMR spectra were analyzed with programs CARA (35) and NMRView (36).

#### Pulsed field gradient (PFG) NMR diffusion measurements

1H NMR diffusion measurements were performed using a stimulated-echo sequence incorporating bipolar gradient pulses (ledbpgppr2s) with diffusion time equal to 60 ms in all experiments with 1mM DNA samples because at higher DNA concentrations, the protein signal was obscured by the DNA signal. The amplitude of the pulsed field gradient varied from 2 to 98% of the maximum value (0.53 T/m) in 32 increments. Data processing and analysis were performed using the Topspin 3.5 Bruker software.

#### NMR structure calculation

3D structures of H19Y were calculated by utilizing internuclear distances from NOESY spectra, dihedral angle restraints generated from the chemical shifts using the program TALOS (37) and hydrogen bond distance restraints using the program XPLOR‐NIH (38). The eight minimum energy structures with no restraint violations were selected from a set of 100 calculated structures as a representative ensemble based on the absence of NOE violations >0.5 Å and dihedral angle violations >5° and assessed for stereochemical quality using the PROCHECK program (39). Experimental restraints and structural statistics are summarized in Supplementary Table S2. Molecules were visualized and aligned using the program PYMOL (40). The coordinates and related information were deposited to Protein Data Bank (PDB code 6URS). Chemical shift information was deposited to Biological Magnetic Resonance Data Bank (BMRB code 30680).

### Microscale thermophoresis

Microscale thermophoresis (MST) experiments were performed using a Monolith NT.115 (NanoTemper) instrument. For protein–DNA binding experiments, Cy5-labeled DR-core, Li or Lo sequences were purchased from IDT (Integrated DNA Technologies, Inc.). For H19Y protein–protein interaction experiments, we used RED-NHS 2nd Generation dye that reacts with primary amines (NanoTemper) and performed labeling at a 1:1 ratio (dye molecule to protein molecule) to have approximately one dye molecule per protein and its location statistically distributed over the protein surface to avoid interference with binding. A total of 40 nM of labeled H19Y or 30 nM of Cy5-labeled DNA was mixed with increasing amounts of unlabeled binding partner, and the mixtures were incubated for 30 min in the dark at room temperature. The experiments were done using Monolith NT.115 premium capillaries. The assay buffer contained 25 mM sodium phosphate buffer at pH 5.2 or pH 7.4, 150 mM NaCl and 0.05% Tween-20 to prevent sample sticking to the capillaries for H19Y experiments. For experiments with full-length SB10 and SB100X transposases, the assay buffer contained 50 mM Tris, 5 mM MgCl_2_, 300 mM NaCl, 2% glycerol, 50 mM Arg-Glu mixture, 1 mM TCEP, 0.1% Triton X-100 and 0.1 mg/ml bovine serum albumin, prepared at pH 7.5. All data were evaluated over the T-jump time interval. Initially, dose response curves obtained from normalized fluorescence (*F*_norm_) were analyzed by the least-squares curve fit using the NanoTemper software. *F*_norm_ is the normalized fluorescence signal, which corresponds to the ratio of the fluorescence value measured in the heated state to the fluorescence value measured in the cold state before the infrared (IR) laser is turned on. Subsequently, the data from different experiment repeats (>3) were analyzed together using Origin 22 software.

### Fluorescence anisotropy and lifetime

Fluorescence anisotropy (FA) and fluorescence lifetime (FLT) measurements were conducted using a custom-built transient fluorometer equipped with a QuadraCentric sample compartment with a cuvette holder (Horiba Scientific), a passively Q-switched microchip YAG laser (SNV-20F-100, 532 nm, 20 kHz, Teem Photonics), a photomultiplier (H6779-20, Hamamatsu) and a digitizer (Acqiris DC252, Agilent). The sample was loaded into a sub-micro cuvette (16.45F-Q-3/Z8.5, Starna Cells, Inc.). Vertically polarized light was used for excitation. The detection arm incorporated either an FF01-582/64 BrightLine single-band bandpass filter or a BLP01-532R EdgeBasic long-pass edge filter (both from Semrock), along with a polarizer. All experiments were conducted at room temperature (21 ± 1°C). The sample fluorescence was digitized after each laser pulse with subnanosecond resolution, typically averaging over a thousand laser pulses. FLT measurements were performed with the polarizer in the detection arm set to the magic angle. FA measurements were conducted by orienting the polarizer vertically (v) and horizontally (h), enabling the measurement of *I*_vv_ and *I*_vh_ components, respectively, of fluorescence intensity excited with vertically polarized light. The instrument response function (IRF) was measured before each experiment using a buffer as the scattering medium. The resulting fluorescence waveform was generally modeled as a two-exponential decay function, A_1_·exp(−*t*/τ_1_) + A_2_·exp(−*t*/τ_2_), convoluted with the IRF, where A_1_ and A_2_ are the amplitudes of the exponential components, and τ_1_ and τ_2_ are the lifetimes of the components.

Data analysis was performed using the FargoFit software package, developed by I. Negrashov (41). FargoFit executes global least-square fitting of multiple time-resolved fluorescence waveforms, allowing for parameter linking between waveforms in different models. The FA of labeled DNA was calculated in the absence or presence of SB transposase variants using the (42)


(3) \begin{equation*}FA = \frac{{{I_{{\rm vv}}} - g \cdot {I_{{\rm vh}}}}}{{{I_{{\rm vv}}} + 2g \cdot {I_{{\rm vh}}}}},\end{equation*}

where the g-factor, determined in a separate experiment, was found to be 1.03 for our setup. The intensity-weighted FLT, τ, of labeled DNA in the absence or presence of SB transposase variants was calculated using the following (42)


(4) \begin{equation*}\tau = \frac{{{A_1}\tau _1^2 + {A_2}\tau _2^2}}{{{A_1}{\tau _1} + {A_2}{\tau _2}}}.\end{equation*}

The integral intensity of the fluorescence was calculated as *A*_1_·τ_1_+*A*_2_·τ_2_, assuming complete fluorescence decay within 50 ns after the laser pulse.

### Protein–DNA docking using PD-DOCK

The PD-DOCK program (43,44) employs a rigid protein–DNA docking procedure and searches for the optimal complex solution using a Monte Carlo algorithm and a knowledge-based, orientation potential for assessing protein–DNA interaction (44). In total, 200 independent docking experiments were carried out to maximize the conformational search space, resulting in a total of 200 predicted protein–DNA complex structures. The interaction energy for each protein–DNA complex was calculated, and expressed in arbitrary units, as a sum of the energies of all residue (*i*) and DNA base (*j*) interactions using our knowledge-based, distance and orientation-dependent protein–DNA interaction potential as shown in Figure 1 (44,45):


(5) \begin{equation*}E = \sum {E_{ij}^0\left( {r,\varphi } \right)} ,\end{equation*}

where *r* and φ represent the distance between residue *i* and base *j* and the angle between residue sidechain and the base plane, respectively.

These complex structures were then clustered hierarchically based on their structural similarity. The distance between two complex structures was the root-mean-square deviation of protein Cα atoms after the corresponding DNA structures were superimposed. The hierarchical clustering was carried out using a complete linkage approach, in which the distance between two clusters is represented by the largest distance among all pairwise distances between the members of two clusters. Using a bottom-up approach and the root-mean-square displacement (RMSD) cutoff of 3 Å, we divided the structures into clusters and ranked the clusters based on the protein–DNA-binding energy of the representative structure, i.e. the complex with the lowest energy within the cluster. The top cluster was selected and used for follow-up analyses.

### Protein–DNA docking using HADDOCK

The structure of the DR-core double helix was generated using the MAKE-NA program (46–49). Chemical shift perturbations were used as ambiguous interaction restraints to drive the docking process using HADDOCK version 2.2 (50). Residues having a weighted-chemical shift perturbation upon binding to DNA >2 SD and displaying high solvent accessibility (>50%) were selected as active residues. Solvent accessibility for the active residues was calculated using the program NACCESS (31). The HADDOCK program was allowed to define passive residues (residues near the interface that may play a role in the formation of the complex) as docking parameters automatically. The lowest energy structure of H19Y calculated with XPLOR-NIH was selected as the starting H19Y structure for the docking. All DNA bases were selected as active bases. During the rigid body energy minimization, 10 000 structures were calculated, and the 200 best solutions based on intermolecular energy were used for the semi-flexible, simulated annealing followed by an explicit water refinement. Docked structures corresponding to the 200 best solutions with lowest intermolecular energies were generated. The 200 solutions were clustered using a 1.0 Å RMSD cutoff criterion into eight different clusters. The clusters were ranked based on the averaged HADDOCK score of their top 10 structures. From these, we selected the top cluster with a *Z*-score of −1.3, a HADDOCK score of −145 ± 7.5 and the most favorable intermolecular energies, to represent the model of the H19Y-DR-core complex.

### Transposition assays

Antibiotic resistance-based transposition assays were done by plating 300.000 HeLa cells per well on a six-well plate one day before transfection and transfecting 100 ng of pT2/HB-puro and 50 ng pFV-SB10, pFV-SB10-H19Y, pFV-SB100X or pFV-SB100X-H19Y in 250 μl MEM by using the Mirus-LT1 transfection reagent (Fisher Scientific). 60% of the transfected cells were seeded on a 10 cm dish 24 h post-transfection with medium containing puromycin. Puromycin-resistant cell colonies were allowed to grow on the dishes for 2 weeks, at which time point they were stained and counted.

## Results

### DNA-binding affinity of the full-length SB transposase

**Figure 2.**
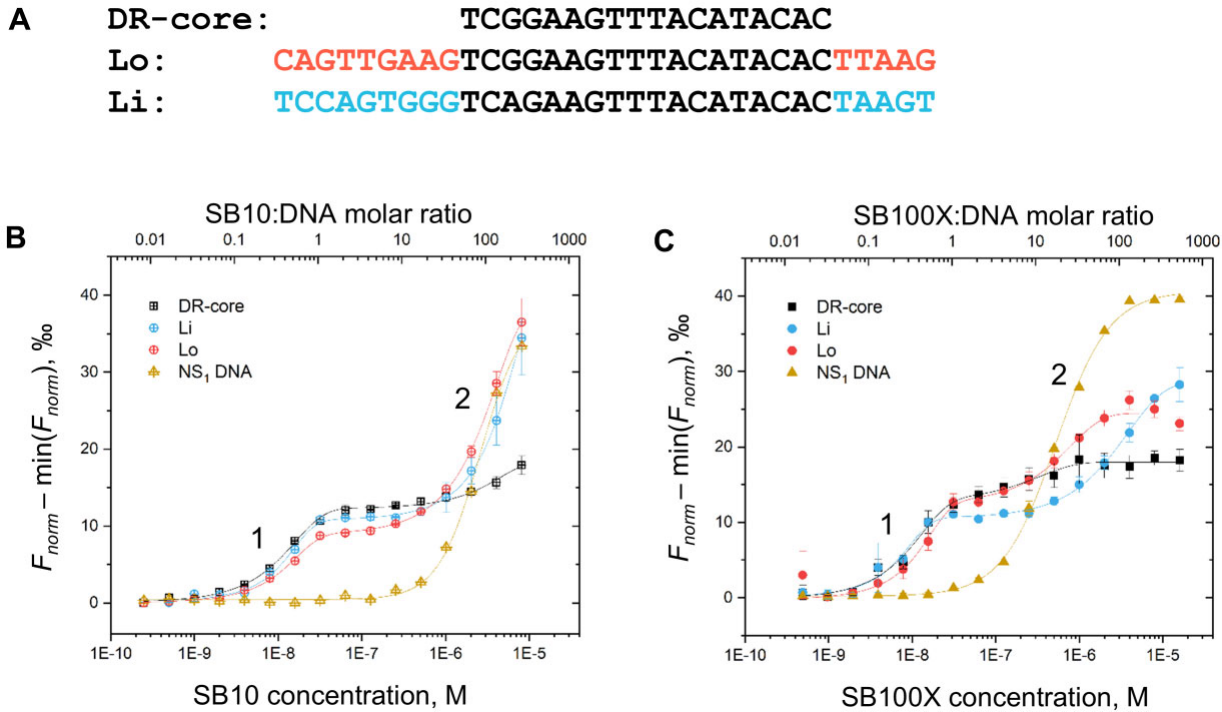
SB10 and hyperactive SB100X transposase binding to the transposon DNA. (**A**) SB transposon DNA sequences of the DR-core, outer (Lo) and inner (Li) transposase-binding sites of the left TIR. Binding affinities for SB10 (**B**) and SB100X (**C**) binding to DR-core, Lo, and Li were evaluated using the MST titration experiment with Cy5-labeled DNA sequences held at a constant concentration of 30 nM, to which unlabeled proteins were added at gradually increasing concentrations. As a control, a Cy5-labeled nonspecific (NS_1_) DNA sequence (5′-ACCTTCCTCCGCAATACTCCCCCAGGT-3′) was used. To facilitate the comparison of binding curves for different DNA sequences we show data on the same scale. For this, we subtracted the respective minimum value of *F*_norm_ for each curve. Experimental error bars show S.E. for *n* ≥ 3 separate experiments. The solid lines represent Hill fits to the experimental data. MST-binding curves reveal two DNA-binding modes, indicated by numbers.

SB transposition begins with the site-specific binding of the SB transposase to the DRs. Because the DNA-binding constants (KDs) of the SB transposase have not been reported, we started with a broad range of protein concentrations of SB10 and hyperactive SB100X full-length transposases and assessed their DNA-binding affinities using Cy5-labeled DR-core or DNA sequences representing the left outer (Lo) and inner (Li) transposase-binding sites (Figure 2A). As a control, we employed a randomly generated Cy5-labeled DNA sequence that is not specifically bound by the SB transposase (NS_1_, 5′-ACCTTCCTCCGCAATACTCCCCCAGGT-3′). Both SB10 and SB100X showed a biphasic binding (Figure 2B and C) to DR-cores, Li, and Lo sequences, and monophasic binding to NS_1_ DNA at near micromolar protein concentrations (protein to DNA molar ratios above 20:1, top axes). The second transition in the biphasic binding to DR-core, Li and Lo sequences occurred at protein concentrations similar to those for NS_1_ DNA, indicating that it likely corresponds to a nonspecific DNA-binding mode. The binding of SB100X to NS_1_ DNA occurred at lower protein concentrations than SB10, indicating that SB100X binds nonspecific DNA with higher affinity than SB10. Calculated equilibrium binding constants (KDs) for NS_1_ DNA were 2.60 ± 0.16 μM for SB10 and 0.56 ± 0.04 μM for SB100X. The first transition in the biphasic binding to DR-core, Li and Lo transposon DNA sequences, occurs at nanomolar binding affinities, at approximately a 1:1 protein to DNA ratio, and is not observed with NS_1_ DNA, thus representing a specific binding of SB10 and SB100X to the transposon DNA sequences. To determine binding constants for specific DNA binding more accurately, we sampled the protein concentration range of the first transition in more detail. Estimated KD values are given in Table 1, and titration curves are shown in Supplementary Figure S1. At 0.05 level significance, SB10 shows a somewhat stronger affinity for binding to Li and Lo, but weaker affinity to DR-core.

**Table 1.** KD values (nM) for full-length SB10 and SB100X transposases binding to DR-core, Li and Lo transposon DNA sequences

Protein/DNA	SB10	SB100X
DR-core	25.3 ± 0.9	17.3 ± 1.1
Li	17.3 ± 1.2	23.6 ± 2.2
Lo	16.6 ± 2.6	23.0 ± 1.9

Previously, we showed that PAI of the SB transposase must fold to bind to the DRs (28,29). Accordingly, we investigated the influence of structural stability of PAI on DNA binding.

H19Y mutation promotes structural stability of the primary DNA-recognition subdomain of the SB transposase

#### Deprotonation of the H19 sidechain drives folding of PAI

**Figure 3.**
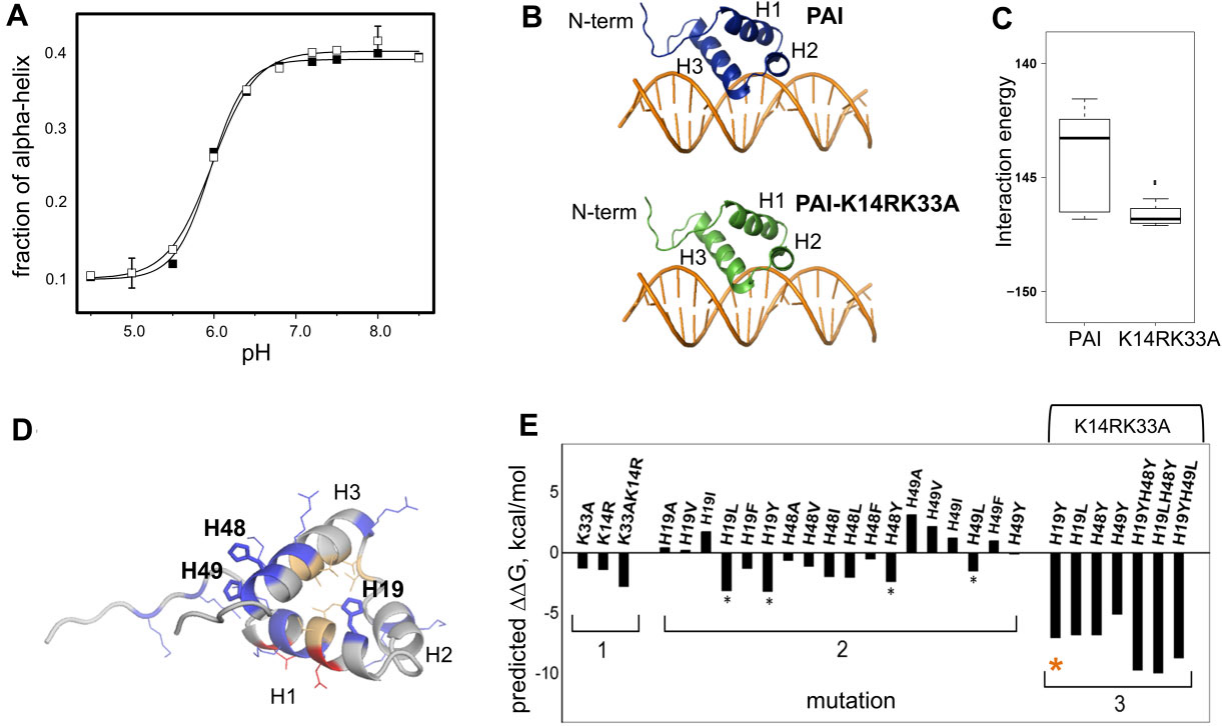
The pH-induced folding of PAI. (**A**) Fraction of the helical conformation versus pH of PAI (filled squares) and PAI-K14RK33A double mutant (open squares) estimated using the DicroWeb server (30,31). Solid lines represent a global sigmoid dose-response fit of the data and are included as a guide to the eye. (**B**) Top PD-DOCK-predicted structures of protein–DNA complexes for PAI subdomain and K14RK33A mutant. (**C**) Interaction energies of PAI subdomain or K14RK33A mutant complexes with DR-core. The K14RK33A mutations provide increased stabilization of protein–DNA complex formation without changing its overall arrangement. (**D**) 3D structure of the PAI-WT subdomain (PDB ID 2M8E). Charged residues are shown in blue (Arg, Lys, His) or red (Asp, Glu). Stick representation highlights histidine residues. Hydrophobic residues surrounding H19 are shown in light orange. Helices H1, H2 and H3 are labeled. (**E**) The predicted effect of mutations on the Gibbs free energy of unfolding of PAI. Group 1 shows the effect of K14R, K33A and K14RK33A mutations. Group 2 shows the effect of H19, H48 and H49 single mutations. Stars label the most energetically favorable substitutions. Group 3 shows the effect of H19, H48 and H49 mutants in the presence of double K14RK33A mutation. The values of ΔΔ*G* were calculated using the Eris protein stability prediction server (54).

Basic pH induces helix formation (Figure 3A; Supplementary Figure S2) of PAI and its double mutant PAI-K14RK33A as in the hyperactive SB100X transposase (Figure 1). The simultaneous presence of K14A and K33A mutations does not alter the pH-dependent folding of PAI. Structural models for complexes formed by PAI and PAI-K14RK33A with the DR-core sequence, generated using the PD-DOCK program shown to produce reliable data without experimental input (43,44,51), are similar (Figure 3B). In both cases, the third helix H3 (residues R36, S37, Q40, T41, R44 K45) interacts with the DNA major groove, whereas helix H1 is positioned away from the DNA. The binding energy of the top predicted protein–DNA complex for PAI-K14RK33A is lower than that for PAI, suggesting a more favorable protein–DNA interaction (Figure 3C). The CD spectra, acquired as a function of pH, show an isodichroic point at ∼204 nm, suggesting a two‐state folding process (Supplementary Figure S2). Fitting MRE values measured at 222 nM with a modified Henderson–Hasselbalch (2) under the assumption of a two-state transition, yields pKa values of 5.98 ± 0.06 and 5.96 ± 0.05 for the PAI and PAI-K14RK33A, respectively (Supplementary Figure S2). These pKa values coincide with the pKa value of histidine sidechain protonation (52,53), indicating that the folding of PAI at basic pH is likely related to the deprotonation of histidine sidechain(s).

PAI contains three histidine residues: H19, H48 and H49 (Figure 3D). Histidines H48 and H49 are located in the C-terminus of PAI on helix H3 and are surface-exposed while H19 is at the end of helix H1, with its sidechain oriented toward the interior of the protein, where it is surrounded by hydrophobic residues I15, L25, V39, I42 and V43 (colored orange in Figure 3D). Therefore, it is plausible to assume that deprotonated H19 promotes PAI subdomain folding. We calculated the changes in the ΔΔG of folding for several H19, H48 and H49 mutants in the absence or presence of the K14RK33A mutations using the Eris protein stability prediction server with the flexible backbone option (54). Group 1 in Figure 3E shows the individual and cumulative favorable effects of K14R and K33A mutations on PAI stability as indicated by negative ΔΔG values. These effects are likely due to a decrease in charge (K33A) and a change in sidechain geometry (K14R) (54,55). Several histidine substitutions lead to increased stability (group 2 in Figure 3E, indicated by stars), in agreement with CD data on the pH-dependence of PAI folding. The effect of histidine substitutions is enhanced in the presence of K14R and K33A (group 3, Figure 3E). Based on the ΔΔG calculations and similarity of DNA binding by PAI and PAI-K14RK33A, we selected the triple mutant K14RH19YK33A (referred to hereinafter as the H19Y) for experimental testing.

**Figure 4.**
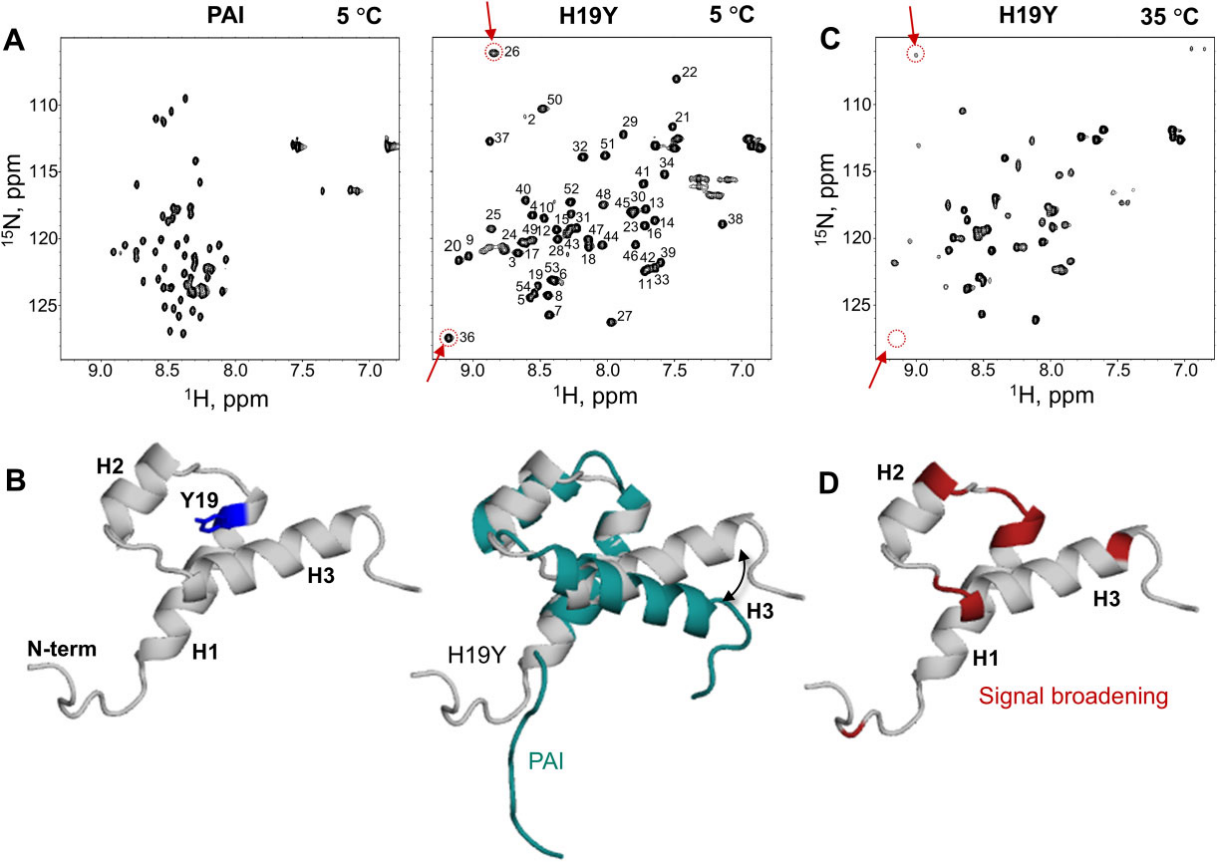
The H19Y mutation eliminates unfolding of PAI. (**A**) The [^1^H,^15^N]-HSQC spectrum of 0.2 mM PAI (left panel) and H19Y (right panel), both collected at pH 5.2 and 5°C. The backbone assignments for H19Y are labeled. (**B**) A representative structure of H19Y from the ensemble of minimal energy structures with Y19 highlighted (left panel) and overlaid with the PAI structure in teal (right panel). The superposition of structures highlights the difference in the orientation of helix H3. (**C**) The [^1^H,^15^N]-HSQC spectrum of H19Y collected at 35°C at pH 5.2. Arrows exemplify the observed peak broadening as compared with 5°C in panel (A). (**D**) The amino acid residues exhibiting significant signal broadening are colored on the H19Y structure.

Figure 4A shows the [^1^H,^15^N]-HSQC spectra of PAI (left panel) and H19Y (right panel), both collected under identical buffer and temperature conditions. The spectrum of PAI displays a very narrow distribution of cross-peaks, with chemical shifts for many of the ^1^H and ^15^N resonances centered around 8 and 120 ppm, indicating that the respective amino acid residues are in a random coil conformation (28,29). The [^1^H,^15^N]-HSQC spectrum of H19Y is drastically different and demonstrates widely dispersed resonances, indicative of a folded structure. This difference is solely due to the H19Y mutation, because PAI-K14RK33A has the same folding properties as PAI (Figure 3A).

#### NMR solution structure of H19Y resembles that of PAI

The left panel of Figure 4B shows a representative H19Y structure selected from the ensemble of lowest energy structures. H19Y folds into a compact, three-helix domain. The PAI (PDB ID 2M8E) and H19Y (PDB ID 6URS) structures are superimposable with an RMSD of 2.6–2.7 Å (Figure 4B, right panel). While the helix H1 is superimposed with a small RMSD difference, the orientation of helix H3 differs between the two structures, likely due to a better fit of the tyrosine sidechain that in contrast to histidine avoids electrostatic repulsion. However, the overall fold of the two structures is similar.

#### Structural stability of H19Y at elevated temperatures

To determine whether H19Y maintains its structure at physiological temperatures, we collected a series of [^1^H,^15^N]-HSQC spectra across 5–45°C temperature range. As the temperature increased, the spectral quality gradually decreased due to signal broadening while the peak dispersion remained unchanged (Figure 4A and C, e.g. see peaks indicated by arrows). [^1^H,^15^N]-HSQC spectra at all temperature increments are provided in the Supplementary Figure S3. The broadened peaks primarily originate from the amino acid residues at or near the DNA-binding site: in the loop region between helices H1 and H2, as well as in the region at the end of the loop between helices H2 and H3, leading into helix H3 (Figure 4D). The peak broadening is not observed in regions thought to be to be involved in protein–protein interactions, as inferred from amino acid sequence analysis and the structures of closely related Mos1 or Tc3 transposases (56,57). MST data collected at pH 5.2 and 7.4 suggest minimal protein association at protein concentrations used in our experiments (Supplementary Figure S4). Using PFG–NMR, we determined that the diffusion coefficient of the H19Y mutant at 35°C was 1.86 ± 0.09 × 10^−10^ m^2^/s, a value that closely matches the theoretically predicted diffusion coefficient of the H19Y monomer, 1.9 × 10^−10^ m^2^/s for a monomer versus 1.3 × 10^−10^ m^2^/s for a dimer, calculated using the HullRad server (58). Thus, although the structure of H19Y is more stable compared with PAI, signal broadening indicates that folding–unfolding still occurs at elevated temperatures.

Transposon DNA-binding mechanism of the primary DNA-recognition subdomain of the SB transposase

#### By promoting the folding of PAI, H19Y replacement favors the formation of protein–DNA complexes

**Figure 5.**
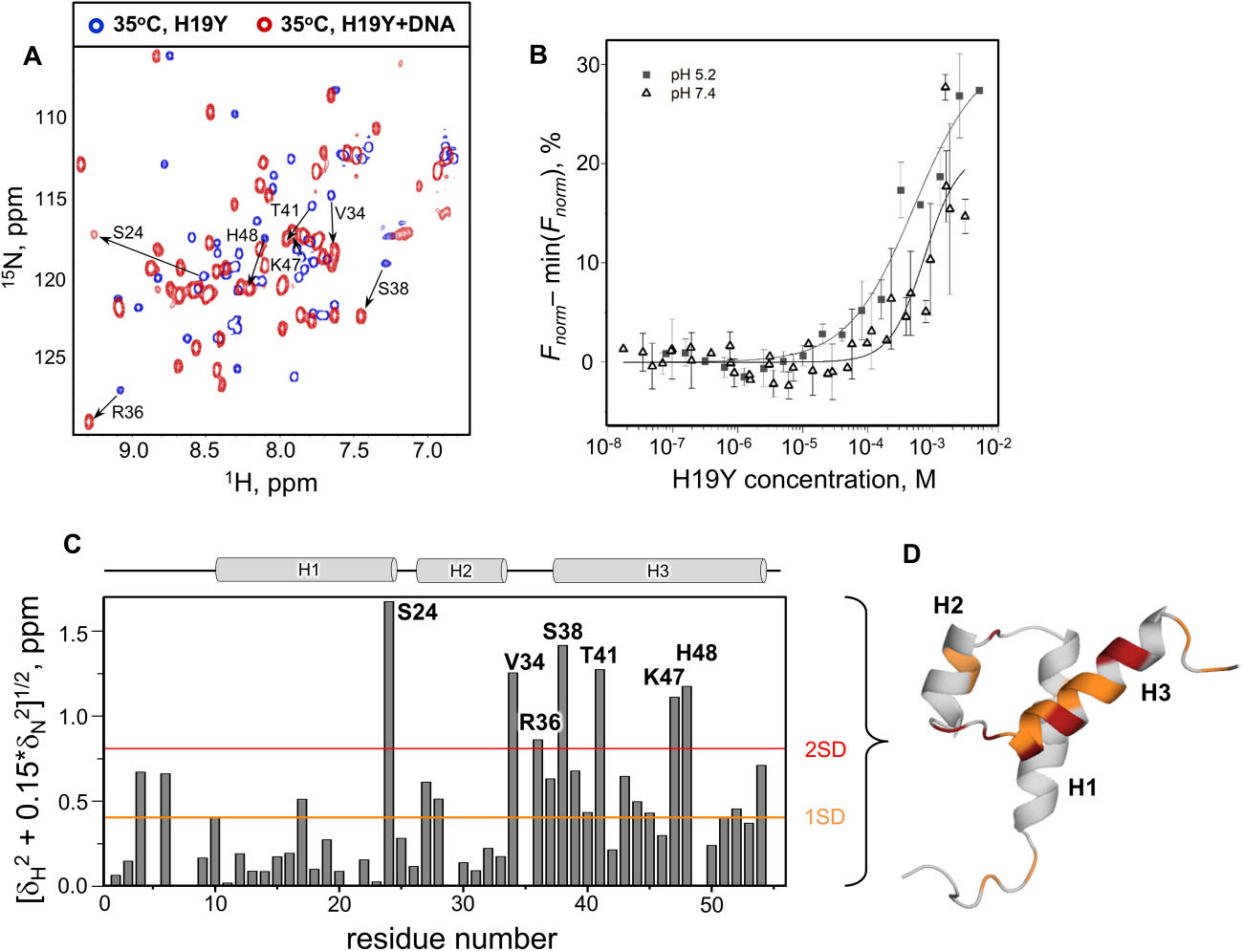
H19Y binding to the transposon DR-core sequence. (**A**) [^1^H,^15^N]-HSQC spectra of 0.085 mM ^15^N,^13^C-labeled H19Y are shown in the absence (blue cross-peaks) and presence (red cross-peaks) of DR-core (1:4.5 molar ratio) collected at 35°C in an aqueous solution of 25 mM sodium phosphate buffer at pH 5.2. Arrows exemplify chemical shift changes caused by the addition of DNA. (**B**) MST-binding curves for H19Y to DR-core collected at pH values of 5.2 or 7.4. Experimental error bars show S.E. for *n* ≥ 3 separate experiments. The solid lines represent Hill fits to the experimental data. (**C**) ^1^H and ^15^N chemical shift differences of H19Y NMR signals from panel (A) due to DR-core binding, weighted according to ((Δδ(^1^H))^2^ + 0.15(Δδ(^15^N))^2^)^1/2^. Orange and red lines represent 1 and 2 standard deviations (SDs) for the data, respectively. (**D**) ^1^H and ^15^N chemical shift differences are colored orange and red according to the magnitude of change (above 1 or 2 SDs, respectively) on the H19Y 3D structure.

Previously, NMR signal broadening, resulting from the intermediate exchange on the chemical shift timescale between folded–unfolded and unbound–DNA–bound states of PAI, prevented a detailed structural analysis of the PAI–DNA complex (28). We assessed the effect of structural stabilization by H19Y on binding the DR-core sequence (the minimal sequence necessary for transposase binding, Figure 2A) using NMR. Whereas, at 5°C, the H19Y mutant alone exhibited the highest structural stability and best quality [^1^H,^15^N]-HSQC spectrum, the amide cross peaks in the presence of DR-core were either missing completely or broadened. However, at 35°C, the intensity of the peaks increased to reveal the [^1^H,^15^N]-HSQC spectrum of the DNA-bound H19Y PAI subdomain (red cross peaks in Figure 5A), demonstrating that by shifting the folding–unfolding equilibrium toward a more structured conformation, the equilibrium between different species was also shifted toward the formation of protein–DNA complexes, in line with the PAI–DNA binding via conformational selection mechanism (29). Remaining signal broadening indicates that the protein–DNA interaction occurs with intermediate-to-fast exchange on the NMR chemical shift timescale and is also partially due to the reduced T2 relaxation time of H19Y in the H19Y-DR-core complex.

Using MST and Cy5-labeled DR-core, we quantitatively assessed the affinity of the H19Y binding to DR-core (Figure 5B). Detecting the fluorescence signal of the Cy5-labeled DR-core allows evaluating the H19Y binding to DNA irrespective of its folding–unfolding dynamics, which would complicate NMR analysis. KD values reveal a sub-millimolar binding affinity of H19Y to DR-core, 0.51 ± 0.29 mM at pH 5.2 and 0.74 ± 0.26 mM at pH 7.4. These values are consistent with the NMR data presented in Figure 5A regarding intermediate-to-fast exchange.

#### Structural model of H19Y-DR-core complex

Despite the overall similarity of peak distribution between the [^1^H,^15^N]-HSQC spectra of H19Y alone and in complex with DNA, there are substantial chemical shifts changes for many residues. To interpret these changes unambiguously, we repeated NMR resonance assignments for the ^15^N^13^C-labeled H19Y in the presence of DNA. The calculated weighted chemical shift perturbations of backbone amide resonances in H19Y upon addition of the DR-core sequence are depicted as a bar graph (Figure 5C) and mapped onto the H19Y structure (Figure 5D). Differences in NMR signal broadening at different temperatures for the DNA-free and DNA-bound [^1^H,^15^N]-HSQC spectra of H19Y necessitated comparing spectra collected at 5°C and 35°C to infer stable and distinct structural states of H19Y. The backbone amide resonances exhibiting significant chemical shift perturbations, exceeding 1 SD (orange) or 2 SD (red), are primarily localized to helix H3 and the loop connecting helices H2 and H3, which is consistent with our hypothesis that helix H3 serves as the DNA-recognition helix of PAI.

**Figure 6.**
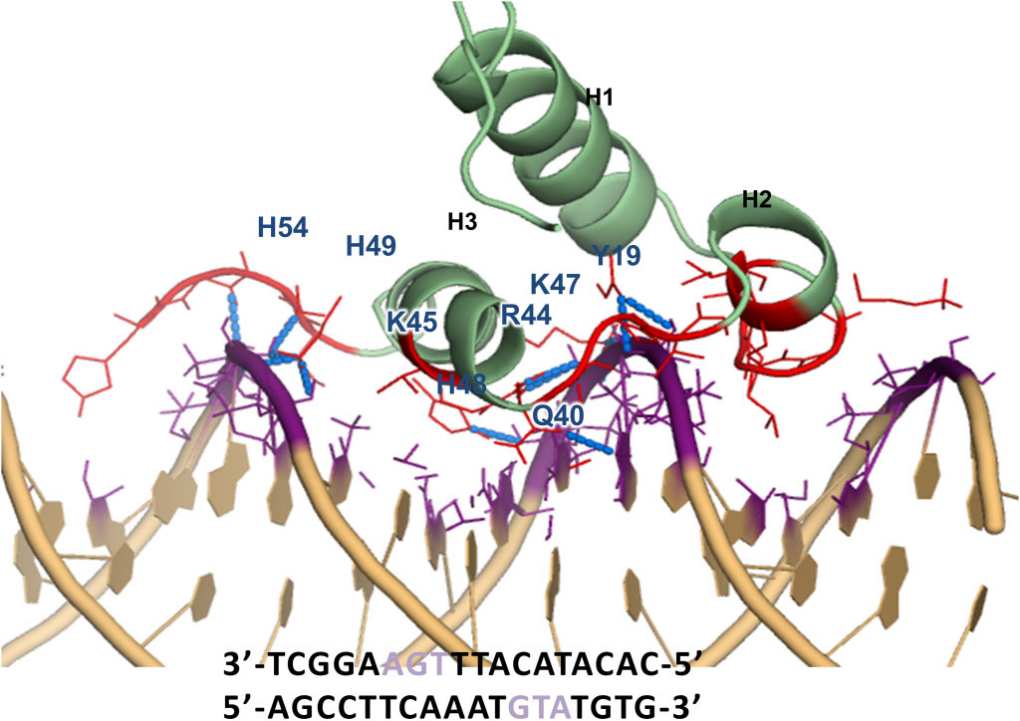
Structural model of H19Y in complex with DR-core constructed using the HADDOCK program. H19Y amino acid residues involved in contact with DR-core are labeled and all interface atoms within 5 Ǻ from the interacting partner are colored in red and purple for H19Y and DR-core, respectively. Dashed blue lines show hydrogen bonds formed between the protein and the DNA.

To gain atomic-level insights into DNA recognition by PAI, we generated structural models of the H19Y-DR-core complex using a molecular docking approach. Intermolecular NOEs for the H19Y-DR-core complex were not detected due to the fast-to-intermediate exchange observed in NMR experiments. Therefore, we utilized the chemical-shift data to construct a structural model of the H19Y-DR-core complex using the HADDOCK program (50). In the top predicted H19Y-DR-core complex (Figure 6), the third helix H3 interacts with the DNA major groove and helix H2 forms contacts with the DNA minor groove as with PAI and PAI-K14RK33A (Figure 3B). Helix H1 is positioned away from DNA, allowing for potential protein–protein interactions. Residues Y19, S23-K30, A33-R36, Q40, T41, R44, K45, K47-H49 and T52-H55 (red in Figure 6) contribute to the interactions with the DNA molecule. The sidechains of Y19, Q40, R44, K45, K47, H48, H49 and H54 form hydrogen bonds with the DNA bases, represented by blue dotted lines.

We also examined the differential binding of H19Y to Li- and Lo-binding sites (Supplementary Figure S5). As with DR-core, the [^1^H,^15^N]-HSQC spectra of the ^15^N-labeled H19Y PAI displayed all signals, corresponding to the DNA-bound conformation of H19Y. The addition of Li DNA sequence induced chemical shift changes distributed throughout the molecule, primarily localized to helices H2 and H3, while a few residues at the end of helix H1 are also affected. The addition of Lo led to significant chemical shift changes in a smaller subset of residues compared with Li, predominantly in helices H2 and H3, but was accompanied by a more pronounced signal broadening, suggesting the difference in binding affinities.

The diffusion coefficients, measured by PFG–NMR for 1 mM Li or Lo addition, were equal to 1.42 ± 0.08 × 10^−10^ and 1.38 ± 0.06 × 10^−10^ m^2^/s, respectively, supporting the formation of monomeric H19Y-Li or H19Y-Lo complexes (59). These data are congruent with the results of the EMSA experiments showing that PAI subdomain binds to the transposon DNA as a monomer (60).

By extension, these findings suggest that the H19Y-DR-core complex is also monomeric and confirm that the observed NMR signal broadening arises from the exchange process between bound and unbound H19Y conformations, as well as between alternative conformational H19Y states. The latter includes conformations sampled by the DNA-free H19Y and the conformational adjustment in H19Y upon DNA binding. Indeed, the structure of the PAI subdomain, particularly the relative orientation of its three helices compared with the DNA-free state, can be stabilized by the interactions of positively charged residues (K30, R36, R44, K45, K47-H49 and H55) at the DNA interface with the negatively charged DNA molecule. This is supported by the reduction in NMR signal broadening, which mainly occurs in the loops connecting the helices in the absence of DNA (compare Figure 4C and D and red cross-peaks in Figure 5A). The stabilization of α-helices coupled to complex formation was previously observed for example in helix H3 of the Pax5 protein (61) that shares structural and amino acid sequence similarity to PAI (62). Our proposed conformational plasticity and selection mechanism is similar to the positive correlation between larger conformational changes upon DNA-binding of transcription factors (63).

### The effect of the structure stabilizing H19Y mutation on the DNA-binding of the full-length SB transposase

Our NMR data clearly show that the H19Y mutation improves DNA-binding properties of PAI by increasing the number of molecules in the DNA-binding competent conformation. However, considering the multi-domain structure of SB transposase, other factors may affect its DNA-binding capability, e.g. other domains contribution to DNA binding or interdomain interactions. Therefore, we investigated the impact of the H19Y-induced stabilization of PAI’s folded conformation on the DNA-binding of the full-length SB10 transposase. Table 2 lists estimated binding constants, and binding curves are provided in Supplementary Figures S6–S8. All three independent techniques (MST, FA and FLT) consistently indicate that the H19Y mutation enhances the binding of SB transposase to the Lo DNA sequence. The binding to Li is improved, but not as strongly, and the binding to DR-core sequence was either weakened insignificantly (MST) or significantly (FA and FLT).

**Table 2.** KD values (nM) for full-length SB10, SB10-H19Y, SB100X and SB100X-H19Y transposases binding to DR-core, Li and Lo transposon DNA sequences

Method	DNA sequence	SB10	SB10-H19Y	SB100X	SB100X-H19Y
MST	DR-Core	25.3 ± 0.9	31.1 ± 2.3	17.3 ± 1.1	31.2 ± 11.1
	Li	17.3 ± 1.2	10.2 ± 0.9	23.6 ± 2.2	10.3 ± 0.8
	Lo	16.6 ± 2.6	11.6 ± 1.8	23.0 ± 1.9	29.7 ± 3.5
FA	DR-Core	37.0 ± 4.0	51.3 ± 8.3	35.1 ± 6.6	104 ± 16
	Li	19.3 ± 2.8	18.2 ± 2.2	27.6 ± 4.9	16.0 ± 3.2
	Lo	23.9 ± 2.8	9.73 ± 2.5	13.7 ± 2.8	17.3 ± 2.2
FLT	DR-Core	29.4 ± 2.0	45.4 ± 4.8	31.6 ± 3.4	117 ± 6.7
	Li	17.2 ± 0.8	16.0 ± 0.7	25.9 ± 2.3	16.2 ± 2.3
	Lo	23.0 ± 2.2	13.2 ± 1.1	16.0 ± 2.6	20.6 ± 0.8

**Figure 7.**
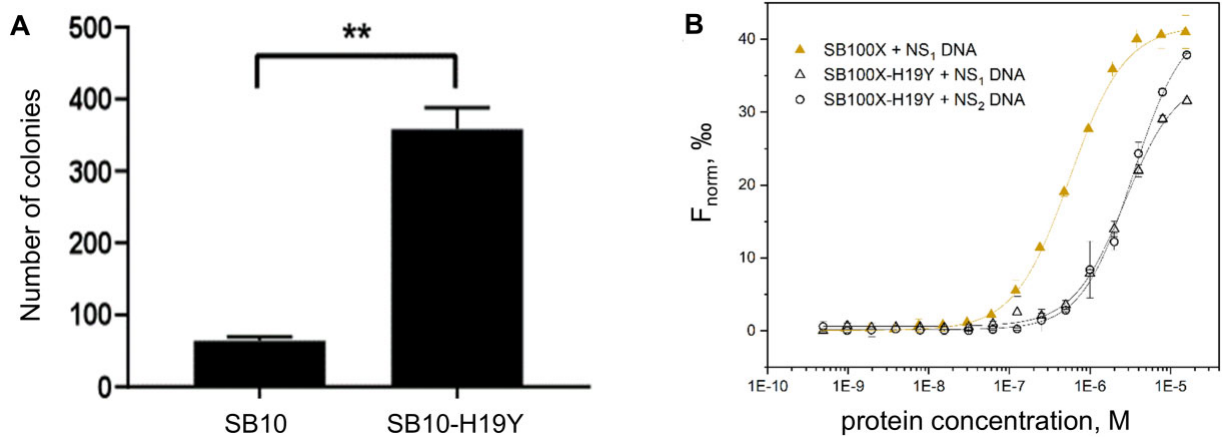
The effect of H19Y mutation on the full-length SB10 transposase activity and SB100X transposase DNA binding. (**A**) SB transposase with the H19Y mutation is hyperactive. Plasmids expressing the first-generation SB transposase SB10 (14) and its derivative containing the H19Y mutation were transiently co-transfected with a puro-tagged transposon donor plasmid into human HeLa cells. The frequency of genomic integration is indicated by the number of puromycin-resistant colonies. Data are represented as mean ± standard deviation, *n* = 3 biological replicates. Asterisks indicate significant differences as determined by Student’s *t*-test **P =0.002. (**B**) MST-binding curves for SB100X and SB100X-H19Y binding to non-specific NS_1_ (5′-ACCTTCCTCCGCAATACTCCCCCAGGT-3′) DNA and NS_2_ DNA (5′-CGGTCTTTCCGTCTT-3′) DNA sequences. Dramatic decrease in nonspecific DNA-binding affinity is observed for SB100X-H19Y mutant. The solid lines represent Hill fits to the experimental data.

To evaluate the impact of the H19Y replacement on the transposition activity of the SB transposase, relative transposition efficiencies were assessed by a colony-forming transposition assay in HeLa cells (14). The analysis of colony formation revealed a >5-fold increase in the numbers of antibiotic-resistant cell colonies obtained with SB10-H19Y compared with SB10 (Figure 7A). We conclude that the enhanced binding ability of SB10-H19Y to the transposon Lo sequence leads to an increase in transposition activity.

We subsequently investigated the impact of the H19Y mutation on the DNA-binding properties of the SB100X hyperactive transposase (Figure 7B) and observed a significant, 6-fold decrease in the binding affinity of SB100X-H19Y to the nonspecific DNA sequence NS_1_ compared with SB100X, with KD values of 2.71 ± 0.21 and 0.56 ± 0.04 μM, respectively. We also tested the binding of SB100X-H19Y to another nonspecific DNA sequence, NS_2_ (5′-CGGTCTTTCCGTCTT-3′), and found the KD value of 3.43 ± 0.24 μM, suggesting a similar trend of weaker binding affinity. This indicates that the H19Y mutation enhances the selectivity of SB100X transposase binding and supports our hypothesis of the conformational selection binding mechanism by PAI.

We then investigated the effect of H19Y mutation on the SB100X transposase binding to the transposon DNA sequences DR-core, Li and Lo (Table 2; Supplementary Figures S6–S8). SB100X-H19Y exhibited stronger binding to Li sequence than SB100X. However, the binding to the DR-core and Lo sequences became weaker. We have also evaluated the transposition efficiency of SB100X-H19Y transposase using a colony-forming transposition assay; however, we did not see an increase in the transposition activity.

## Discussion

Previously, we showed that the PAI DNA-binding subdomain of SB transposase must adopt a compatible (folded) conformation to bind the transposon DNA (29). However, this conformation forms most efficiently under non-physiological conditions (28). We hypothesized that stabilizing this folded conformation of PAI under physiological conditions would enhance transposition. Given PAI’s net positive charge, the electrostatic repulsion among positively charged amino acid residues may destabilize its structure. To replicate the putative active conformation of PAI, we introduced specific amino acid substitutions to enhance its structural stability. Two hyperactive mutations, K14R and K33A (15), did not affect PAI folding. However, we found that the pH-induced transition to the active state of PAI occurred at pH corresponding to the transition of histidine imidazole sidechain from cationic to neutral (52,53). We surveyed several histidine substitutions and generated a triple PAI mutant (K14R, H19Y and K33A), named H19Y, with enhanced structural stability compared to PAI. The 3D structures of PAI (28) and H19Y are similar. But, in contrast to PAI, H19Y produced tractable NMR [^1^H,^15^N]-HSQC spectra in the presence of the DR-core sequence for building a structural model of the H19Y-DR-core complex. This structure showed that helices H3 and H2 and the turn connecting these helices participate in binding to the DRs with helix H3 being the primary DNA-recognition helix, substantiating our prior predictions (14,23,28). The contribution of L25 and R36 to DNA binding (24) further supports our model.

By utilizing H19Y, surprisingly, we found a rather weak binding affinity between PAI and the DR-core sequence with KD values within the sub-millimolar range. The experiments using longer DNA sequences, both Li and Lo, suggest a stronger binding affinity as the observed NMR signal broadening is increased, shifting the exchange regime from fast toward intermediate. The relatively weak DNA-binding affinity of the primary SB DNA-binding domain suggests a cooperation among multiple transposase domains for enhanced DNA-binding affinity. Indeed, our data show nanomolar DNA-binding affinity for both full-length SB10 and SB100X, likely because a modular organization of the SB transposase allows for the formation of stable and structurally optimal nucleoprotein complex, needed for successful transposition. To the best of our knowledge, we present the first quantitative estimation of the DNA-binding constants for the full-length SB transposase. Our determined KD values are comparable to those reported for another mariner transposase SETMAR, 85 nM determined by EMSA (64) and 53 ± 4 nM by measuring the FA (65). We anticipate that the insight into the DNA-binding properties of SB10 and SB100X transposases provided by our data may facilitate the search for optimal experimental conditions for cryo-EM structural studies of the SB nucleoprotein complex.

The comparison of SB10 and SB10-H19Y full-length transposases binding to DR-core, Li, and Lo sequences showed that H19Y increased its DNA-binding affinity to Lo sequence where the transposon is excised, which is in line with our predictions that structural stability of PAI enhances DNA-binding properties of SB transposase, which may lead to enhanced transposition activity (Figure 7A). While we observed a significant increase in DNA-binding affinity of SB100X transposase with the H19Y structurally stabilized PAI subdomain to the Li DNA sequence, we observed weakened affinity to the DR-core and Lo DNA sequences. We also did not observe an increase in the transposition activity as with SB10 transposase. It is possible that the effect is masked by an already high, 100-fold increase in the transposition activity of SB100X transposase via other mechanisms, including the increased DNA-binding affinity due to the K33A mutation or the need for stronger binding to the Lo sequences (24). This observation signifies the dependence of SB transposition on several factors, with DNA-binding being only one of them, and explains the effectiveness of molecular evolution approach, which optimizes function by simultaneously screening mutations with different underlying molecular mechanisms, to design hyperactive transposase (15).

While investigating full-length SB transposase binding to the transposon DNA-binding sites DR-core, Li and Lo, we discovered the bimodal DNA binding of both SB10 and SB100X transposases, with a second binding mode corresponding to non-specific DNA binding. Non-specific binding can be expected due to the high positive charge of SB transposase (14). In the case of SB100X transposase, stabilizing PAI structure with H19Y mutation significantly improved its DNA-binding selectivity (Figure 7B), which agrees with the idea of conformational selection transposon DNA binding.

The property of being disordered in the DNA-unbound form and folding upon DNA binding is commonly observed in protein–nucleic acid interactions. In the absence of a preformed interface, a protein can rapidly scan the surfaces of the DNA target and adopt a folded conformation upon binding to a specific DNA sequence (66,67). In the case of SB transposase, its PAI subdomain binds the transposon DNA via a pre-folded conformation. This suggests that, in the rational engineering of efficient transposases, modifications to PAI should follow the general principle of increasing its structural stability. We also propose that increasing the selectivity and specificity of DNA binding for targeted gene delivery can be applied to the SB transposase, despite first direct fusions of target-specific DNA-binding domains to SB transposase diminished the SB transposition activity (68,69). To achieve this, the selection of the target DNA sequence should begin with the DNA site cleaved by the catalytic domain and ensuring an optimal spacing to the site where the engineered PAI subdomain variant would bind specifically. This parallels earlier observations that highlight the paramount importance of selecting an appropriate target site for successful SB transposition (70).

## Supplementary Material

gkae1188_Supplemental_File

## Data Availability

The data underlying this article are available in the article and in its online supplementary material. Atomic coordinates have been deposited in the RCSB Protein Data Bank under accession number 6URS. Chemical shift assignments were deposited to the BioMagResBank (BMRB) under accession number 30680. Any additional data underlying this article will be shared upon reasonable request to the corresponding author.
