# Supplementary material for: DNA binding and transposition activity of the Sleeping Beauty transposase: role of structural stability of the primary DNA-binding domain
Source: Nucleic Acids Res. 2024 Dec 9;53(2):gkae1188. doi: 10.1093/nar/gkae1188 (PMC11754664; doi:10.1093/nar/gkae1188)
Supplement: gkae1188_Supplemental_File [file gkae1188_supplemental_file.pdf]

Supplementary Material.

Supplementary Table S1. Nomenclature and amino acid sequences of SB transposase variants used in this study with H19Y mutation highlighted in orange and hyperactive mutations of SB100X highlighted in blue.

Name	Mutations	Amino acid sequence
**PAI**		MGKSKEISQDLRKKIVDLHKSGSSLGAISKRLKVPRSSVQTIVRKYKHHGTTQHH
**PAI-K14RK33A**	K14R<br>K33A	MGKSKEISQDLRKRIVDLHKSGSSLGAISKRLAVPRSSVQTIVRKYKHHGTTQHH
**PAI-H19Y**	H19Y	MGKSKEISQDLRKKIVDLYKSGSSLGAISKRLKVPRSSVQTIVRKYKHHGTTQHH
**H19Y**	K14R<br>H19Y<br>K33A	MGKSKEISQDLRKRIVDLYKSGSSLGAISKRLAVPRSSVQTIVRKYKHHGTTQHH
**SB10**		MGKSKEISQDLRKKIVDLHKSGSSLGAISKRLKVPRSSVQTIVRKYKHHGTTQPSYRSGRRRVLSPR<br>DERTLVRKVQINPRTTAKDLVKMLEETGTKVSISTVKRVLYRHNLKGRSARKKPLLQNRHKKARLRF<br>ATAHGDKDRTFWRNVLWSDETKIELFGHNDHRYVWRKKGEACKPKNTIPTVKHGGGSIMLWGCFAAG<br>GTGALHKIDGIMRKENYVDILKQHLKTSVRKLKLGRKWVFQMDNDPKHTSKVVAKWLKDNKVKVLEW<br>PSQSPDLNPIENLWAELKKRVRARRPTNLTQLHQLCQEEWAKIHPTYCGKLVEGYPKRLTQVKQFKG<br>NATKY

**SB10-H19Y**	H19Y	MGKSKEISQDLRKKIVDL**Y**KSGSSLGAISKRLKVPRSSVQTIVRKYKHHGTTQPSYRSGRRRVLSPR<br>DERTLVRKVQINPRTTAKDLVKMLEETGTKVSISTVKRVLYRHNLKGRSARKKPLLQNRHKKARLRF<br>ATAHGDKDRTFWRNVLWSDETKIELFGHNDHRYVWRKKGEACKPKNTIPTVKHGGGSIMLWGCFAAG<br>GTGALHKIDGIMRKENYVDILKQHLKTSVRKLKLGRKWVFQMDNDPKHTSKVVAKWLKDNKVKVLEW<br>PSQSPDLNPIENLWAELKKRVRARRPTNLTQLHQLCQEEWAKIHPTYCGKLVEGYPKRLTQVKQFKG<br>NATKY
**SB100X**	K14R<br>K33A<br>R115H<br>RKEN214DAVQ<br>M243H<br>T314N	MGKSKEISQDLRK**R**IVDLHKSGSSLGAISKRL**A**VPRSSVQTIVRKYKHHGTTQPSYRSGRRRVLSPR<br>DERTLVRKVQINPRTTAKDLVKMLEETGTKVSISTVKRVLYRHNLKG**H**SARKKPLLQNRHKKARLRF<br>ATAHGDKDRTFWRNVLWSDETKIELFGHNDHRYVWRKKGEACKPKNTIPTVKHGGGSIMLWGCFAAG<br>GTGALHKIDGIM**DAVQ**YVDILKQHLKTSVRKLKLGRKWVFQ**H**DNDPKHTSKVVAKWLKDNKVKVLEW<br>PSQSPDLNPIENLWAELKKRVRARRPTNLTQLHQLCQEEWAKIHP**N**YCGKLVEGYPKRLTQVKQFKG<br>NATKY
**SB100X-H19Y**	K14R<br>K33A<br>R115H<br>RKEN214DAVQ<br>M243H<br>T314N	MGKSKEISQDLRK**R**IVDL**Y**KSGSSLGAISKRL**A**VPRSSVQTIVRKYKHHGTTQPSYRSGRRRVLSPR<br>DERTLVRKVQINPRTTAKDLVKMLEETGTKVSISTVKRVLYRHNLKGHSARKKPLLQNRHKKARLRF<br>ATAHGDKDRTFWRNVLWSDETKIELFGHNDHRYVWRKKGEACKPKNTIPTVKHGGGSIMLWGCFAAG<br>GTGALHKIDGIM**DAVQ**YVDILKQHLKTSVRKLKLGRKWVFQ**H**DNDPKHTSKVVAKWLKDNKVKVLEW<br>PSQSPDLNPIENLWAELKKRVRARRPTNLTQLHQLCQEEWAKIHP**N**YCGKLVEGYPKRLTQVKQFKG<br>NATKY

Supplementary Table S2. Structural statistics for the NMR structure of the H19Y

**Restraints and statistics**	
**Restraints**	
NOE distance restraints (total)	557
intra-residue (j–i = 0)	302
sequential (j–i = 1)	145
medium range (j–i = 2)	22
medium range (j–i = 3)	50
medium range (j–i = 4)	15
long range (j–i ≥ 5)	23
Hydrogen bonds	28
TALOS derived dihedral angle restraints	66
**Violations**	
NOE distance violations >0.3 Å	0
Dihedral angle violations >5°	0
**RMS deviation from mean structure (Å)**	
Backbone atoms	0.75
**Ramachandran statistics** (backbone, ordered residues, ensemble of 10 structures)	
Analyzed	352/456 (77%)
Favored	330 (94%)
Allowed region	22 (6%)
Outliers	0 (0%)

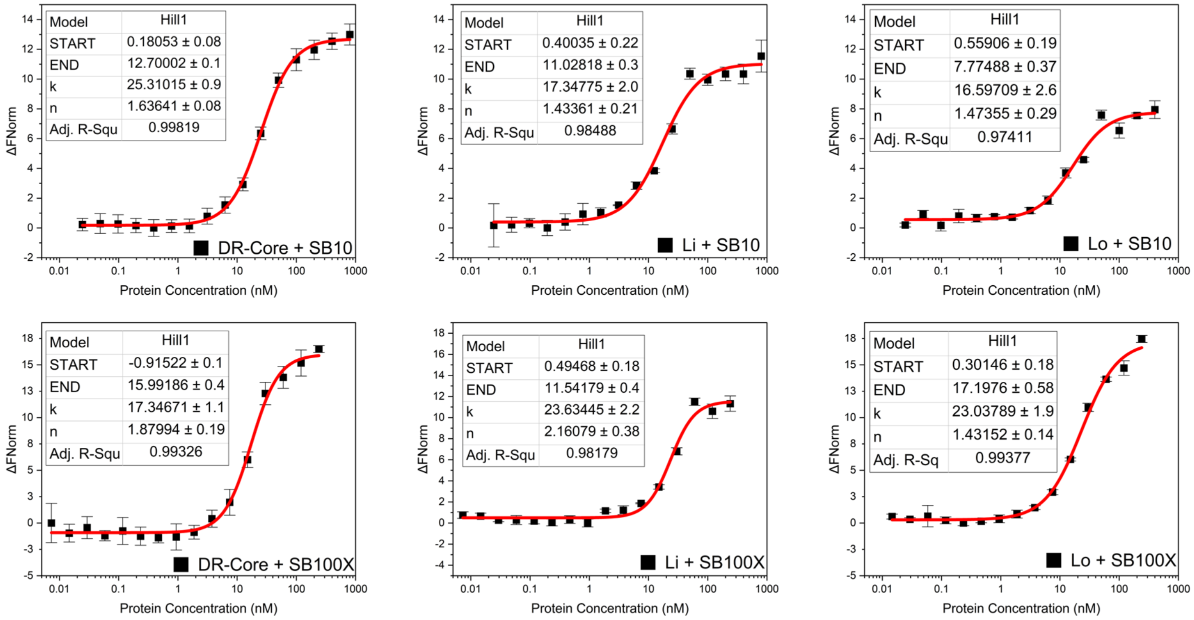

Supplementary Figure S1. The MST data for SB10 and SB100X full-length transposases binding to DNA. The solid lines represent dose-response fits of the experimental data using the Hill function. The curves are averaged over n ≥ 3 independent experiments, with error bars representing S.E..

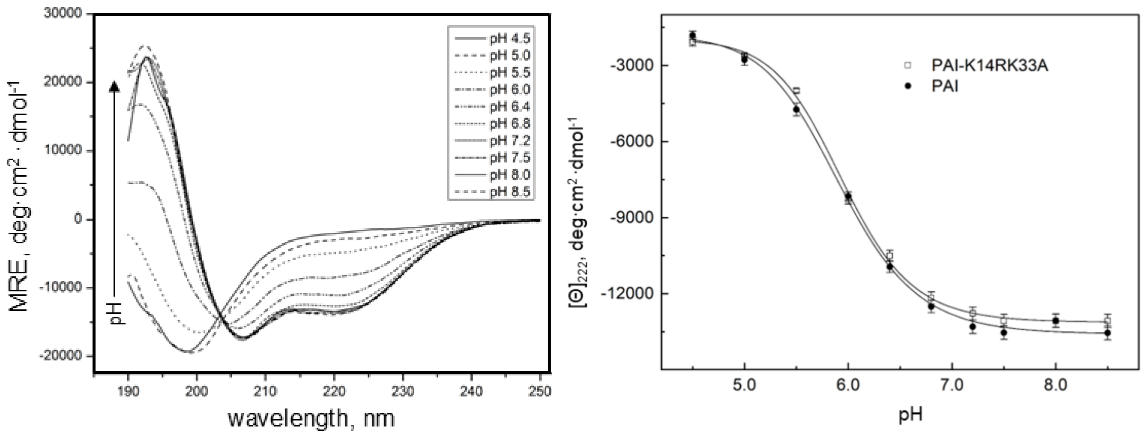

Supplementary Figure S2. (Left panel) The pH-induced folding of the PAI subdomain. (A) Far UV CD spectra (190-260 nm) of PAI subdomain collected in 25 mM aqueous sodium phosphate buffer at solution pH between 4.5 and 8.5. Increasing the pH induces alpha-helical structure as evidenced by a gradual increase in negative peak intensities at 208 and 222 nm, and a positive peak intensity at 192 nm. (Right panel) The dependence of $[\theta]_{222}$ on solution pH is shown for the PAI subdomain (solid squares) and K14RK33A mutant (open squares). Solid lines represent the sigmoid dose-response fit of experimental data using Eq. 2. The p*Ka* values yielded by these fits are equal to 5.98 ± 0.06 and 5.96 ± 0.05 (S.E.), respectively.

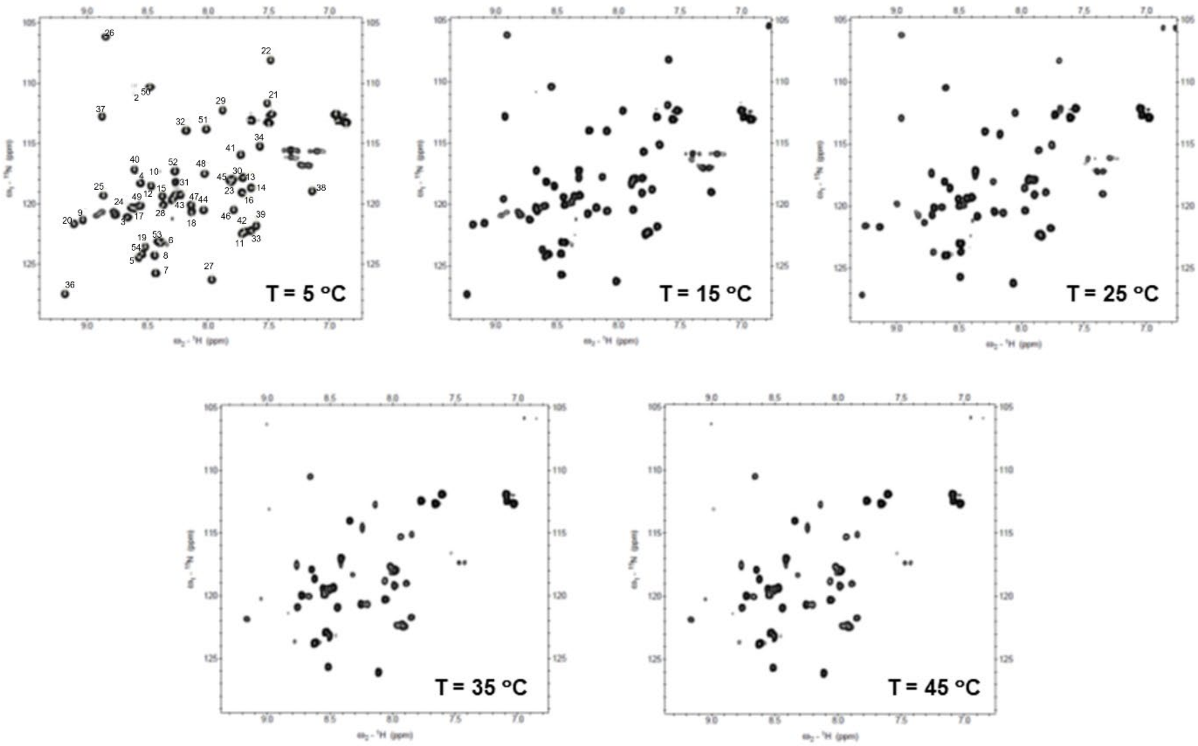

Supplementary Figure S3. The [^{1}H,^{15}N]-HSQC spectra of the H19Y mutant collected at 5, 15, 25, 35, and 45 °C at pH 5.2.

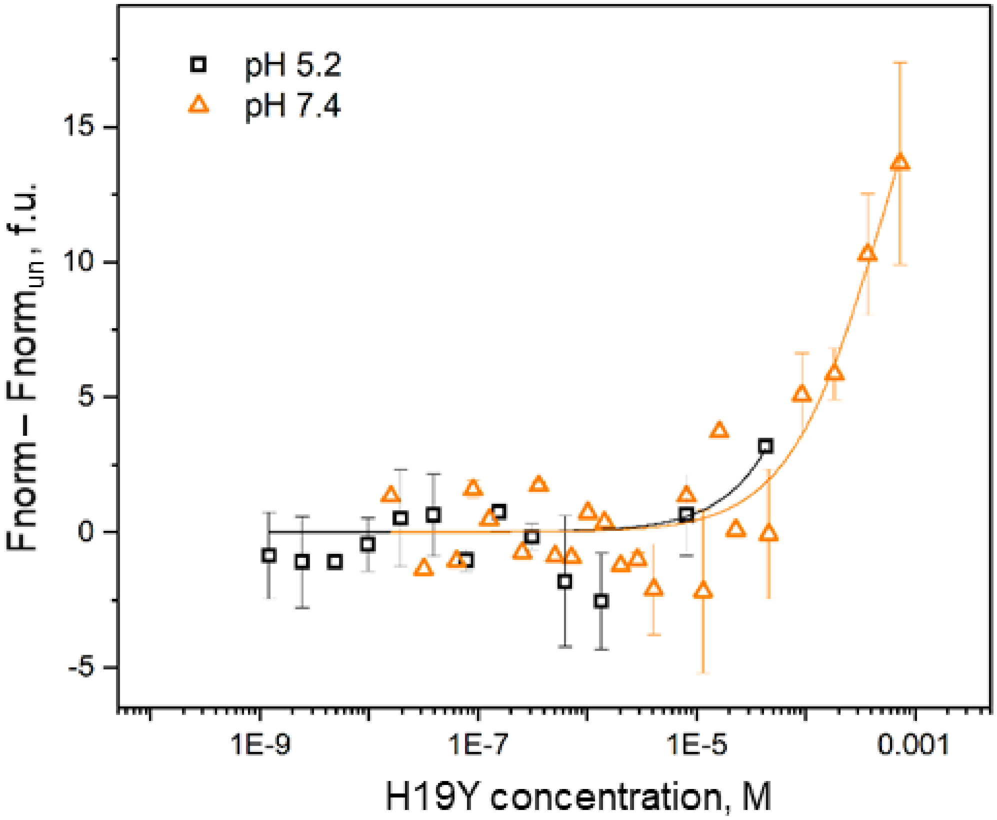

Supplementary Figure S4. The MST data for the H19Y mutant were obtained at pH 5.2 and 7.4 and a temperature of 35 °C. The solid lines depict dose-response fits of the experimental data using the Hill function. The high-concentration plateau could not be reached due to induced inside the capillaries significant sample aggregation at millimolar concentrations, revealed by the bumps on the MST traces. This aggregation effect was more pronounced at pH 5.2. Our observations indicate that we did not observe H19Y dimerization or higher order oligomerization at the concentrations utilized in NMR experiments. Furthermore, the dimerization constant of the H19Y mutant is estimated to be ~ 0.5 ± 0.3 mM or greater at pH 7.4.

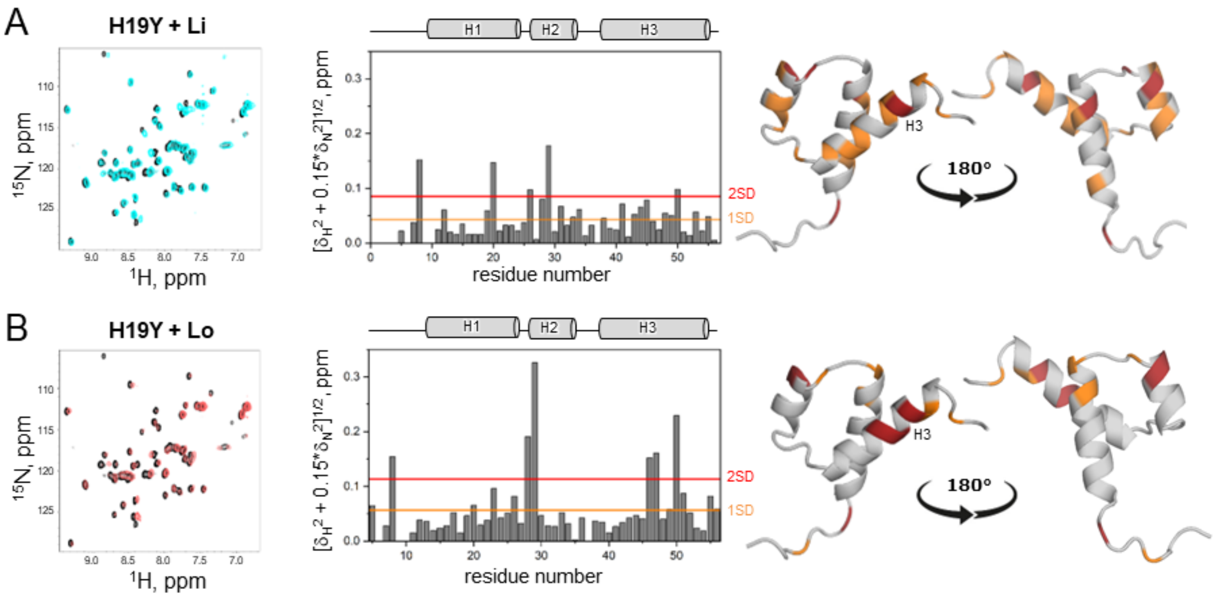

Supplementary Figure S5. H19Y binding to the transposon Li and Lo sequences. (A) DNA binding to Li sequence. [^{1}H,^{15}N]-HSQC spectra of 0.085 mM ^{15}N,^{13}C-labeled H19Y is shown in the absence (black cross-peaks) and presence (cyan cross-peaks) of Li (1:5 molar ratio) collected at 35 °C in an aqueous solution of 25 mM sodium-phosphate buffer at pH 5.2. (B) DNA binding to Lo sequence. [^{1}H,^{15}N]-HSQC spectra at pH 5.2 of 0.085 mM ^{15}N,^{13}C-labeled H19Y is shown in the absence (black cross-peaks) and presence (red cross-peaks) of Lo (1:5 molar ratio) collected at the same conditions as Li.

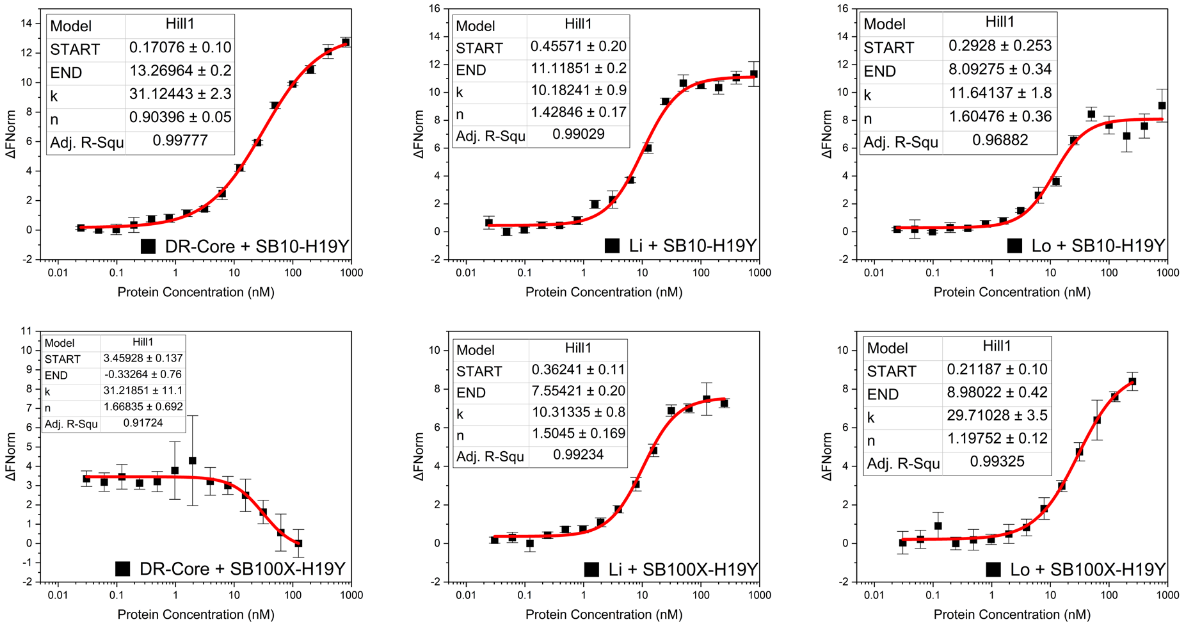

Supplementary Figure S6. The MST data for the SB10-H19Y and SB100X-H19Y binding to DR-core, Li, and Lo. Experimental error bars show S.E. for n ≥ 3 separate experiments. The solid lines represent Hill fits to the experimental data.

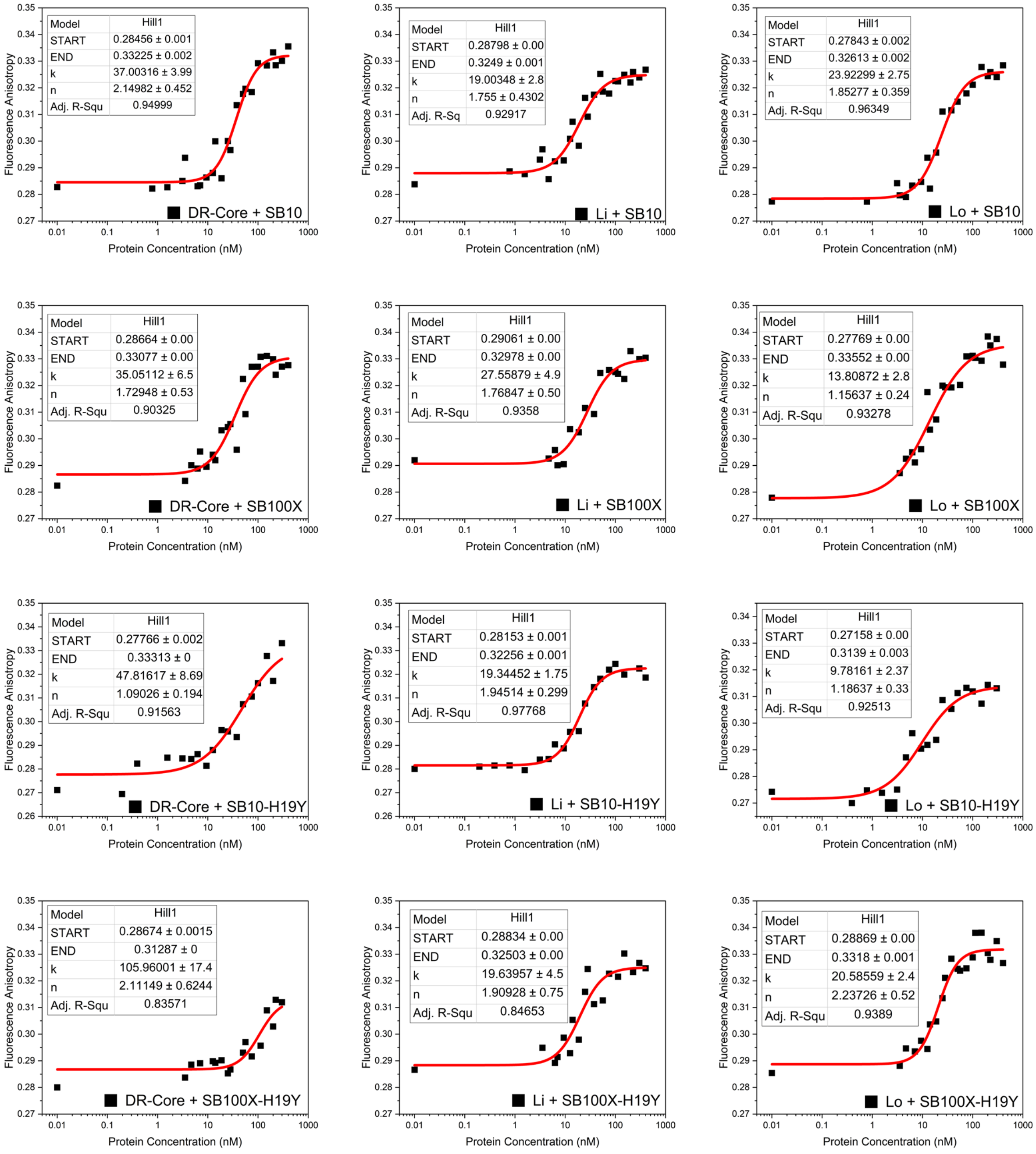

Supplementary Figure S7. The fluorescence anisotropy data for SB10, SB10-H19Y, SB100X, and SB100X-H19Y full-length transposases binding to DNA. The solid lines represent dose-response fits of the experimental data using the Hill function.

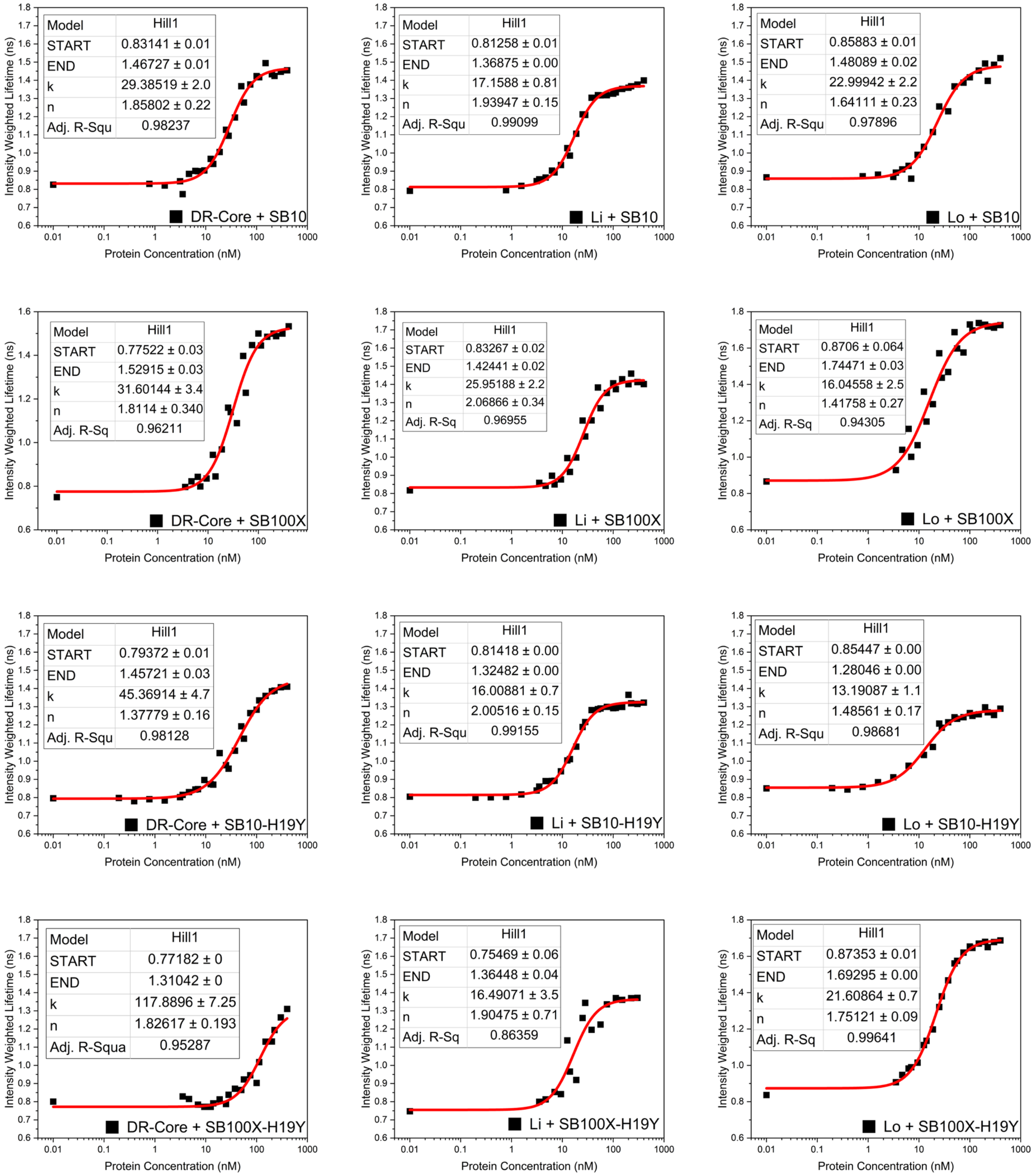

Supplementary Figure S8. Representative fluorescence lifetime binding curves for SB10, SB10-H19Y, SB100X, and SB100X-H19Y full-length transposases binding to DNA. The solid lines represent dose-response fits of the experimental data using the Hill function.
